# Supplementary material for: Rates of SARS-CoV-2 transmission and vaccination impact the fate of vaccine-resistant strains
Source: Sci Rep. 2021 Jul 30;11:15729. doi: 10.1038/s41598-021-95025-3 (PMC8324827; doi:10.1038/s41598-021-95025-3)
Supplement: Supplementary file 1 — Supplementary Figures. [file 41598_2021_95025_MOESM1_ESM.pdf]

Rates of SARS-CoV-2 transmission and vaccination impact the fate of vaccine-resistant strains

Simon A. Rella[1], Yuliya A. Kulikova[2], Emmanouil T. Dermitzakis[3*], Fyodor A. Kondrashov[1*]

[1]*Institute of Science and Technology Austria, 1 Am Campus, Klosterneuburg, 3400, Austria*

[2]*Banco de España, Calle Alcala 48, 28014 Madrid, Spain*

[3]*Department of Genetic Medicine and Development, University of Geneva Medical School, Geneva, Switzerland*

*Corresponding authors, ETD Emmanouil.Dermitzakis@unige.ch, FAK fyodor.kondrashov@ist.ac.at

Supplementary Figures and Legends

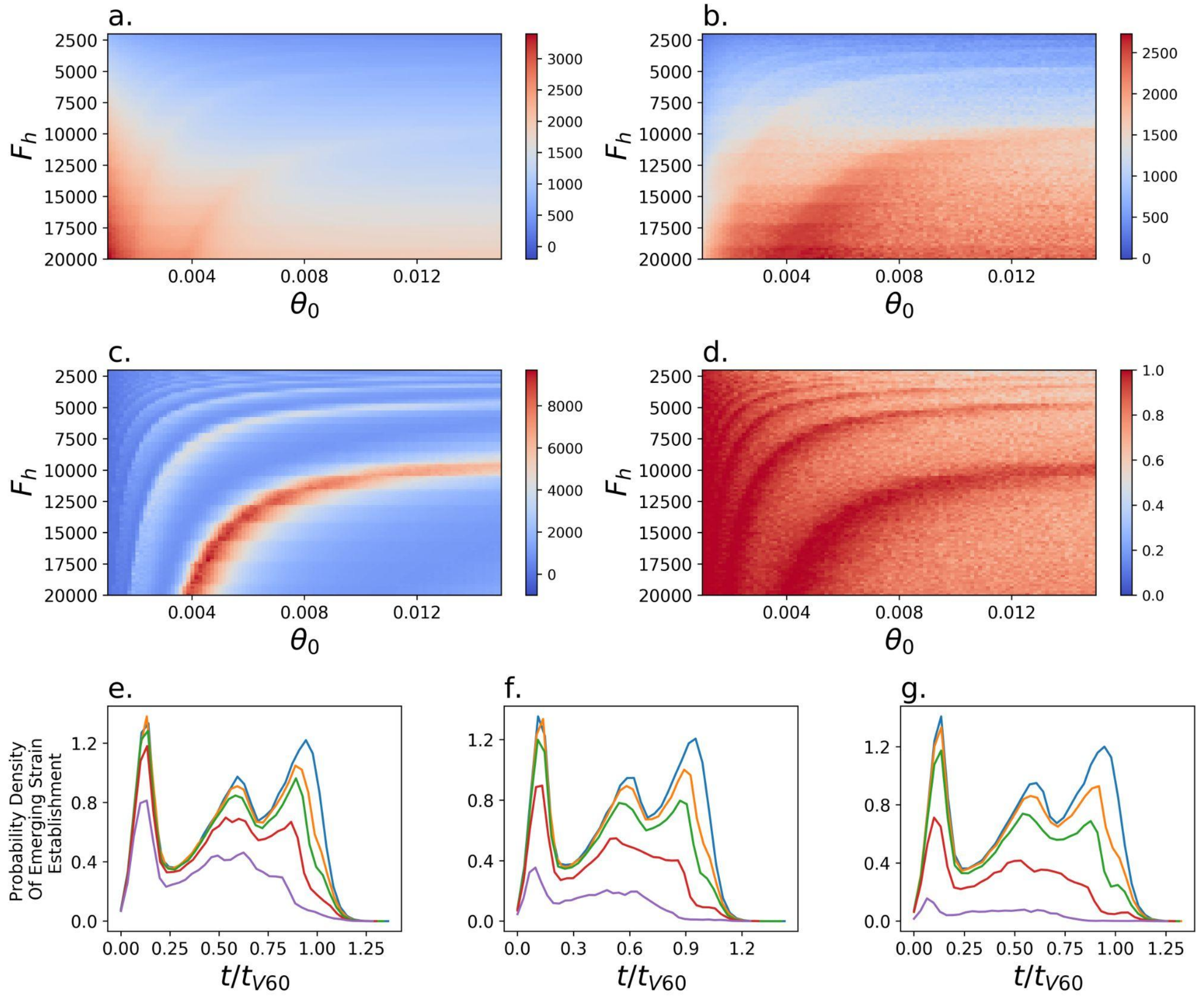

Figure S1 Impact of the rate of vaccination and initiation of low rate of transmission on model dynamics for p = 10^{-5}. The cumulative death rate from the **a**, wildtype and **b,** resistant strains, **c**, the number of wildtype-strain infected individuals at t_{v60}, the point in time when 60% of the population is vaccinated and **d**, the probability of resistant strain establishment. **e-g,** Probability density that the resistant strain emerges as a function of time since the start of the simulation, *t*, rescaled by the time at which 60% of the individuals are vaccinated, t_{v60}, summed across simulations with θ (0.001 through 0.015), F_h (2,000 through 20,000). The impact of the extraordinary low transmission period centered at $t/t_{v60} = 1$ on the likelihood of emergence of the resistant strain as a function of the duration of that period, *T* (colour-coded), and the intensity of the reduction of transmission **e**, $\beta_l = 0.055$, **f**, $\beta_l = 0.03$, **g**, $\beta_l = 0.01$.

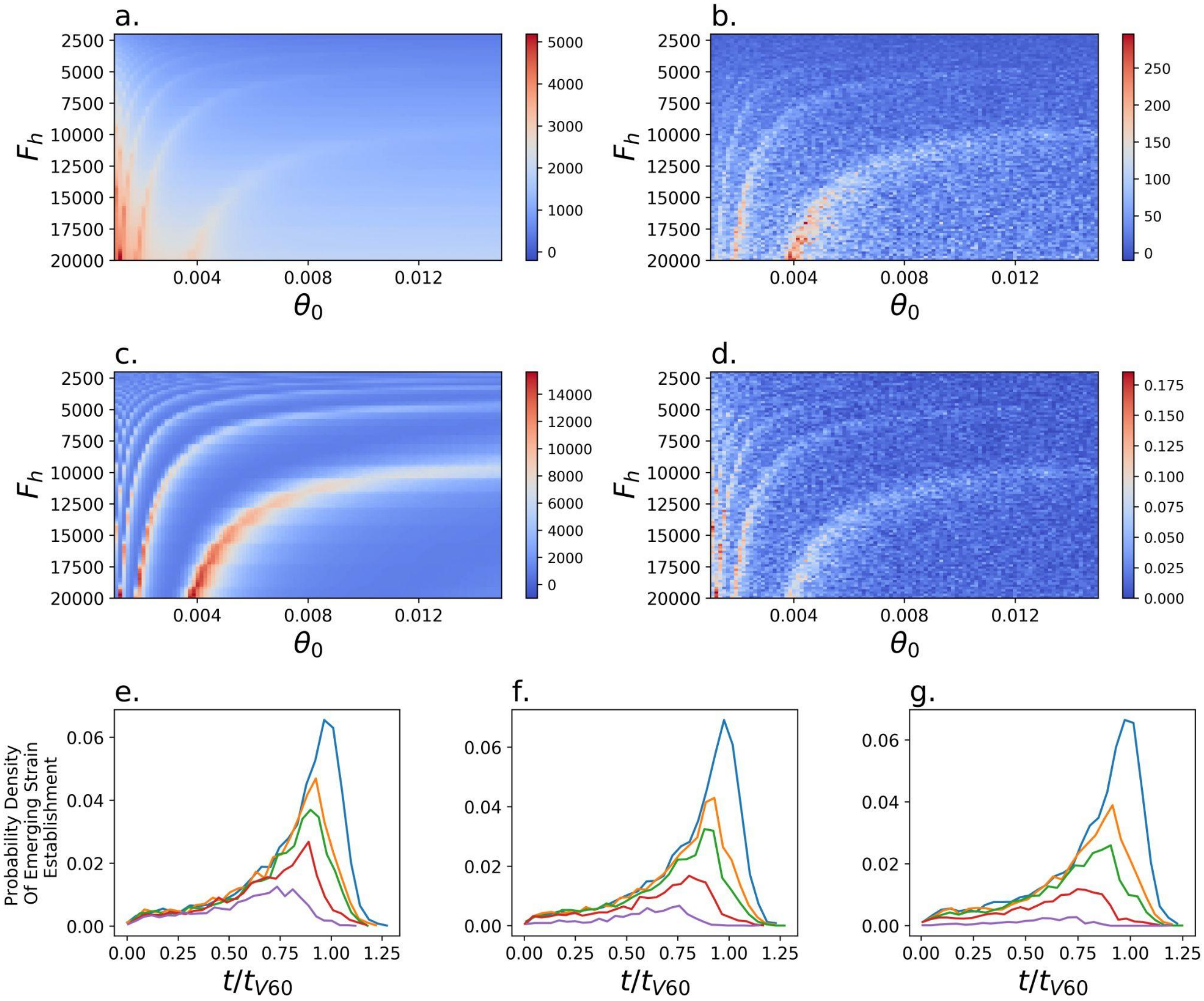

Figure S2 Impact of the rate of vaccination and initiation of low rate of transmission on model dynamics for p = 10^{-7}. The cumulative death rate from the **a**, wildtype and **b,** resistant strains, **c**, the number of wildtype-strain infected individuals at t_{v60}, the point in time when 60% of the population is vaccinated and **d**, the probability of resistant strain establishment. **e-g,** Probability density that the resistant strain emerges as a function of time since the start of the simulation, t, rescaled by the time at which 60% of the individuals are vaccinated, t_{v60}, summed across simulations with θ (0.001 through 0.015), F_h (2,000 through 20,000). The impact of the extraordinary low transmission period centered at $t/t_{v60} = 1$ on the likelihood of emergence of the resistant strain as a function of the duration of that period, T (colour-coded), and the intensity of the reduction of transmission **e**, $\beta_l = 0.055$, **f**, $\beta_l = 0.03$, **g**, $\beta_l = 0.01$.

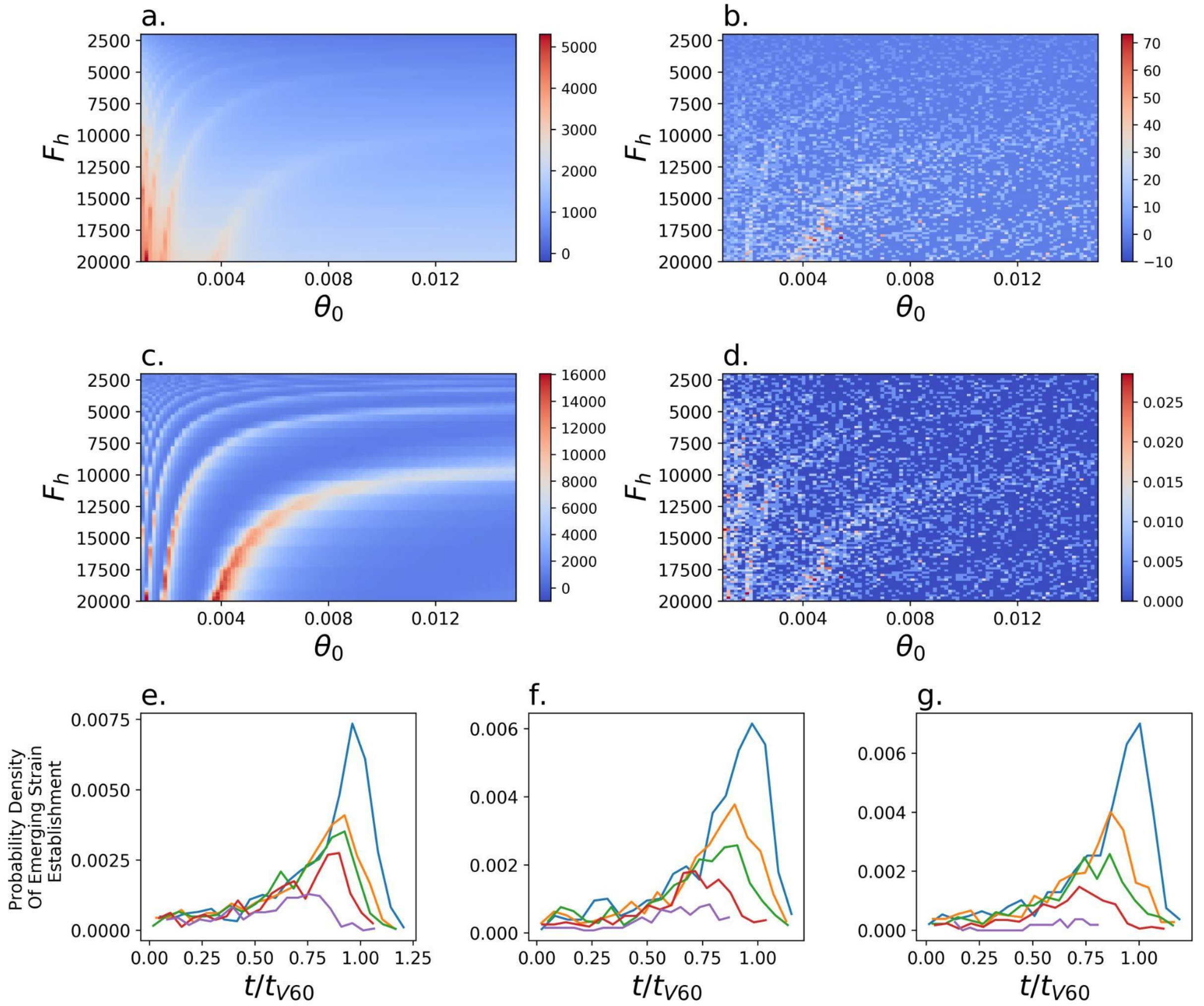

Figure S3 Impact of the rate of vaccination and initiation of low rate of transmission on model dynamics for p = 10^{-8}. The cumulative death rate from the **a**, wildtype and **b,** resistant strains, **c**, the number of wildtype-strain infected individuals at t_{v60}, the point in time when 60% of the population is vaccinated and **d**, the probability of resistant strain establishment. **e-g,** Probability density that the resistant strain emerges as a function of time since the start of the simulation, t, rescaled by the time at which 60% of the individuals are vaccinated, t_{v60}, summed across simulations with θ (0.001 through 0.015), F_h (2,000 through 20,000). The impact of the extraordinary low transmission period centered at $t/t_{v60} = 1$ on the likelihood of emergence of the resistant strain as a function of the duration of that period, T (colour-coded), and the intensity of the reduction of transmission **e**, $\beta_l = 0.055$, **f**, $\beta_l = 0.03$, **g**, $\beta_l = 0.01$.

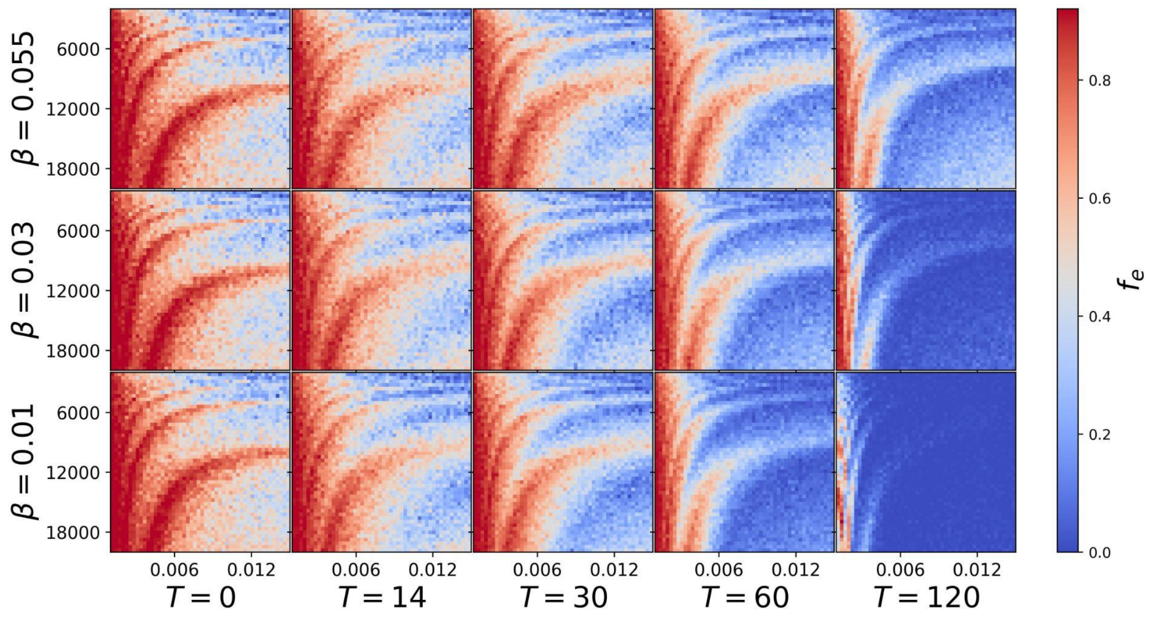

Figure S4 The probability of establishment of the resistant strain for p = 10^{-5}. The influence of low transmission period centered at $t/t_{v60} = 1$ on probability of establishment of the resistant strain as a function of the duration of that period, T, and the intensity of the reduction of transmission, β.

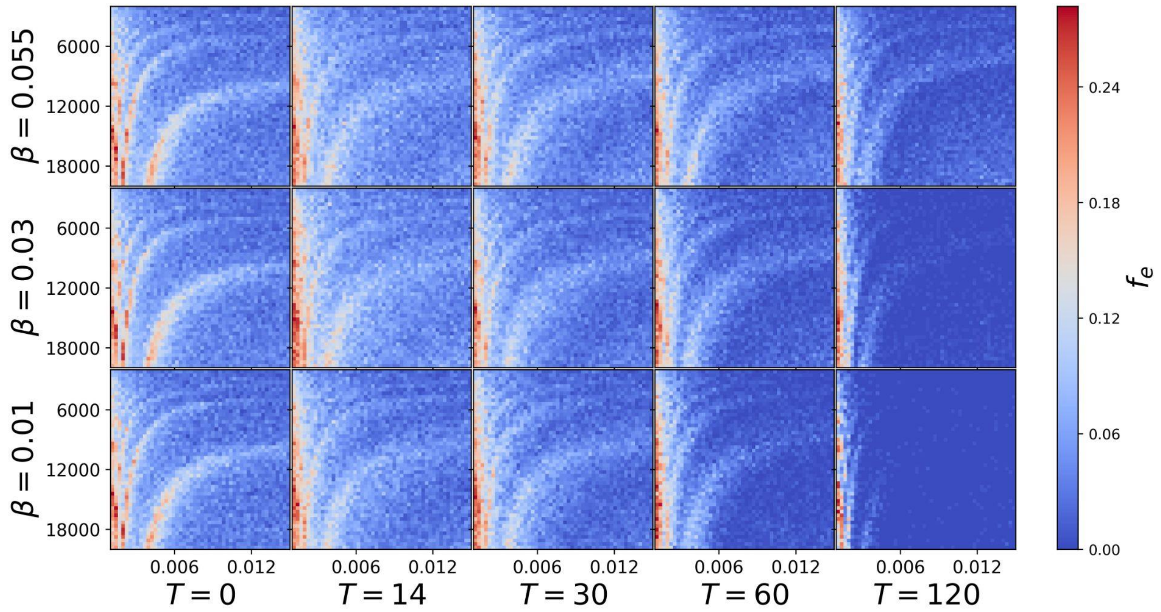

Figure S5 The probability of establishment of the resistant strain for p = 10^{-6}. The influence of low transmission period centered at $t/t_{v60} = 1$ on probability of establishment of the resistant strain as a function of the duration of that period, T, and the intensity of the reduction of transmission, β.

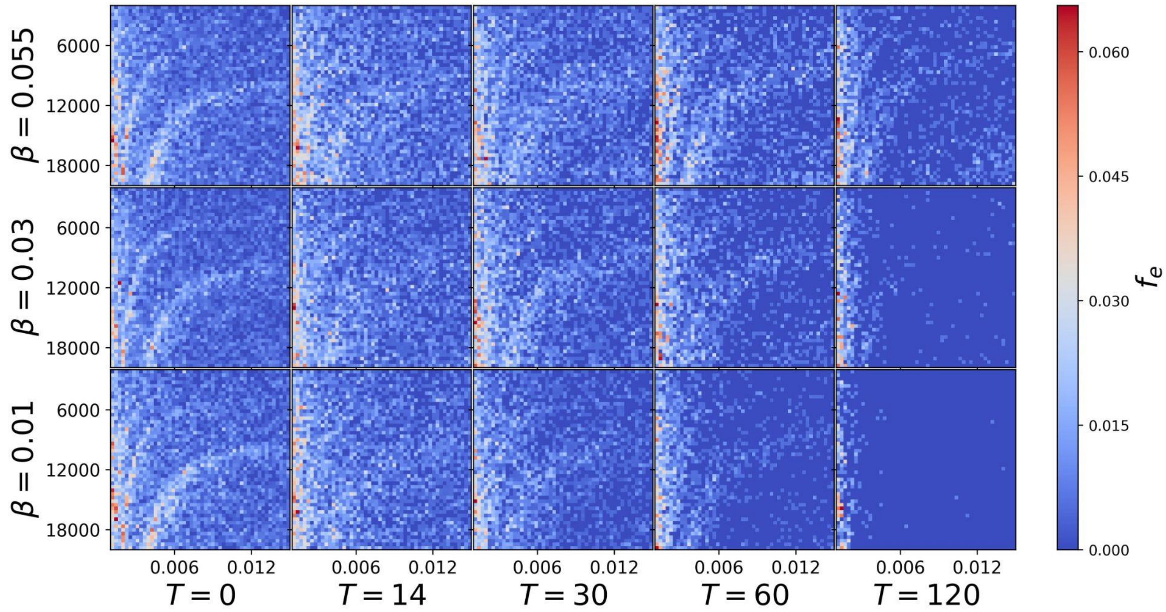

Figure S6 The probability of establishment of the resistant strain for p = 10^{-7}. The influence of low transmission period centered at $t/t_{v60} = 1$ on probability of establishment of the resistant

strain as a function of the duration of that period, T, and the intensity of the reduction of transmission, β.

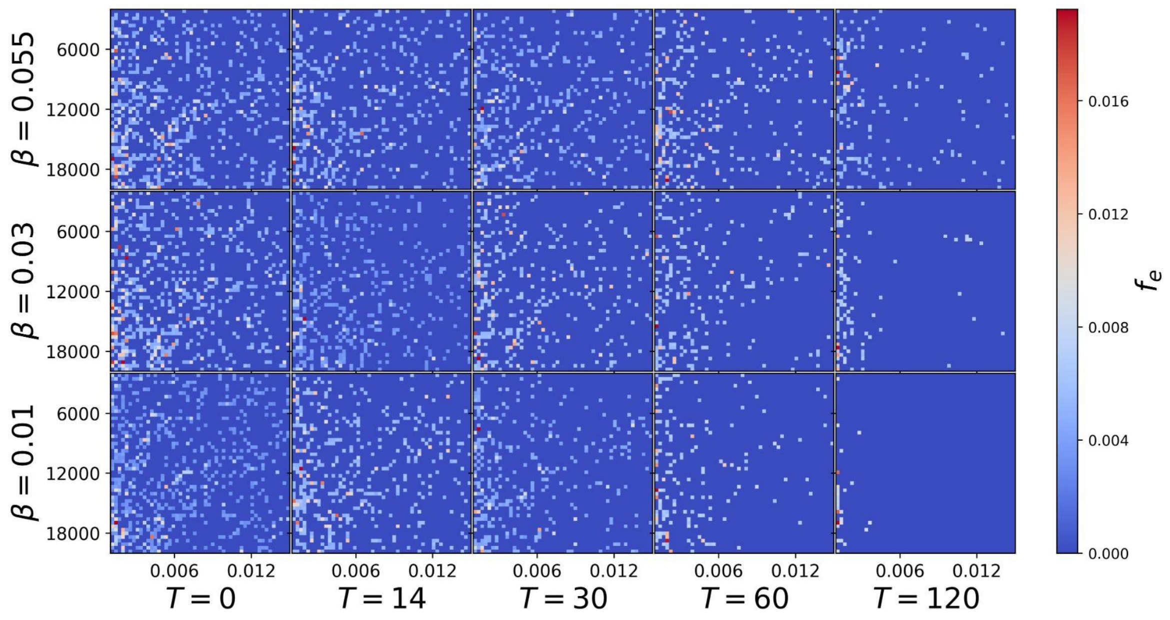

Figure S7 The probability of establishment of the resistant strain for p = 10^{-8}. The influence of low transmission period centered at $t/t_{v60} = 1$ on probability of establishment of the resistant strain as a function of the duration of that period, T, and the intensity of the reduction of transmission, β.

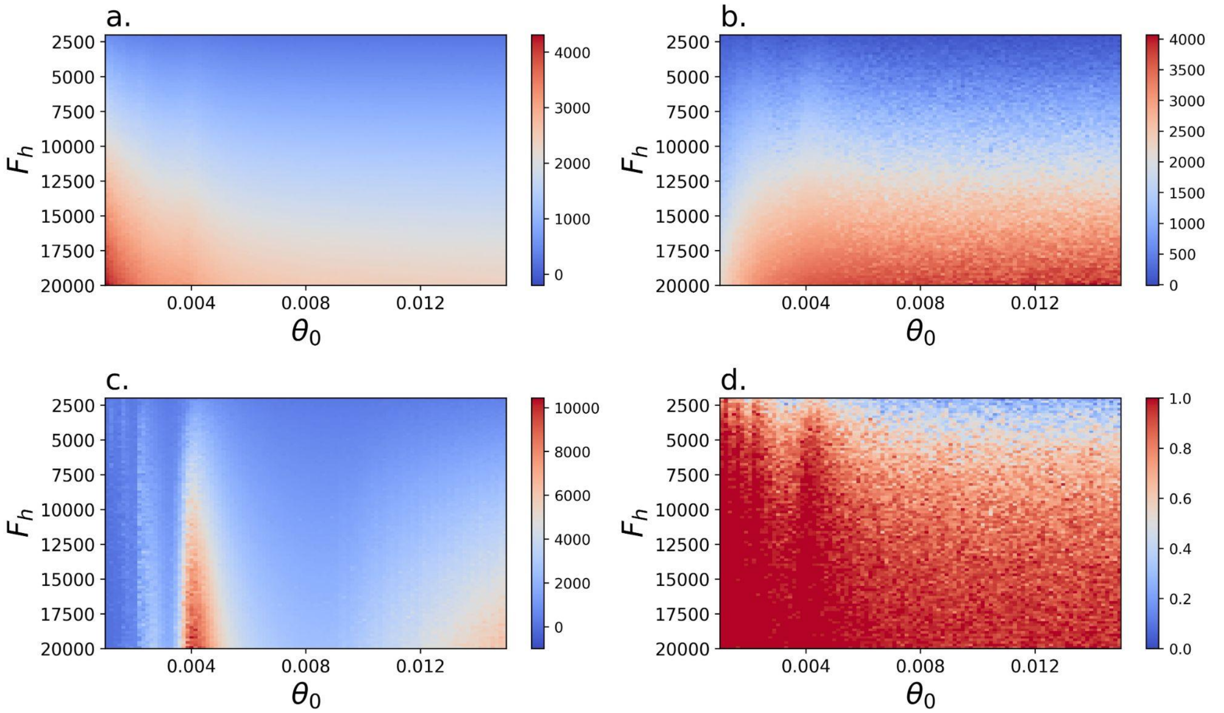

Figure S8 Impact of the rate of vaccination and initiation of low rate of transmission on model dynamics for p = 10^{-5} and exit from low transmission at F_l=F_h/8. The cumulative death rate from the **a**, wildtype and **b,** resistant strains, **c**, the number of wildtype-strain infected individuals at t_{v60}, the point in time when 60% of the population is vaccinated and **d**, the probability of resistant strain establishment.

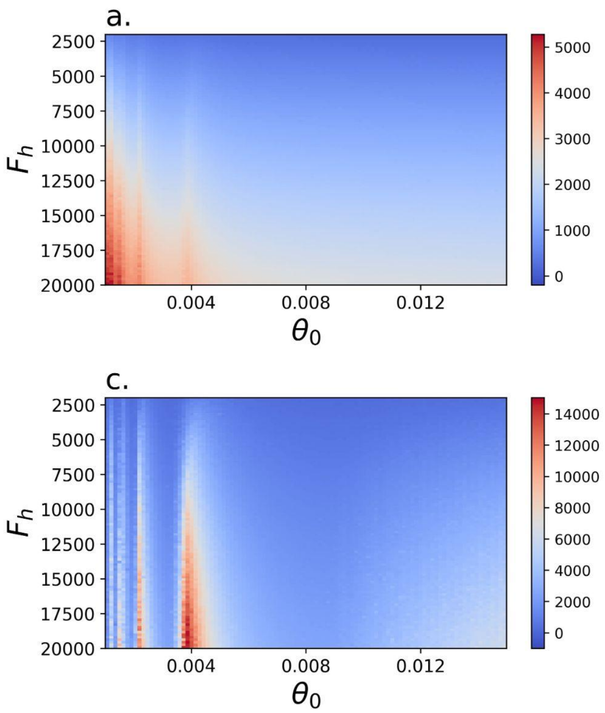

Figure S9 Impact of the rate of vaccination and initiation of low rate of transmission on model dynamics for p = 10^{-6} and exit from low transmission at F_l=F_h/8. The cumulative death rate from the **a**, wildtype and **b,** resistant strains, **c**, the number of wildtype-strain infected individuals at t_{v60}, the point in time when 60% of the population is vaccinated and **d**, the probability of resistant strain establishment.

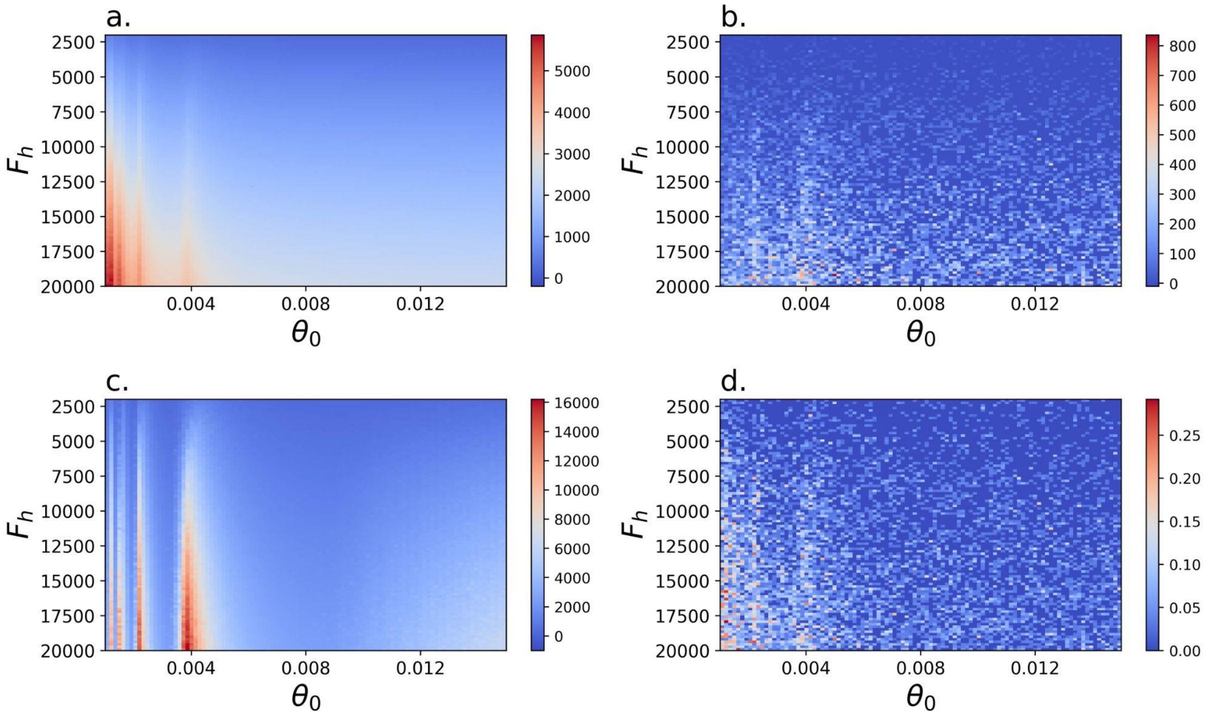

Figure S10 Impact of the rate of vaccination and initiation of low rate of transmission on model dynamics for p = 10^{-7} and exit from low transmission at F_l=F_h/8. The cumulative death rate from the **a**, wildtype and **b,** resistant strains, **c**, the number of wildtype-strain infected individuals at t_{v60}, the point in time when 60% of the population is vaccinated and **d**, the probability of resistant strain establishment.

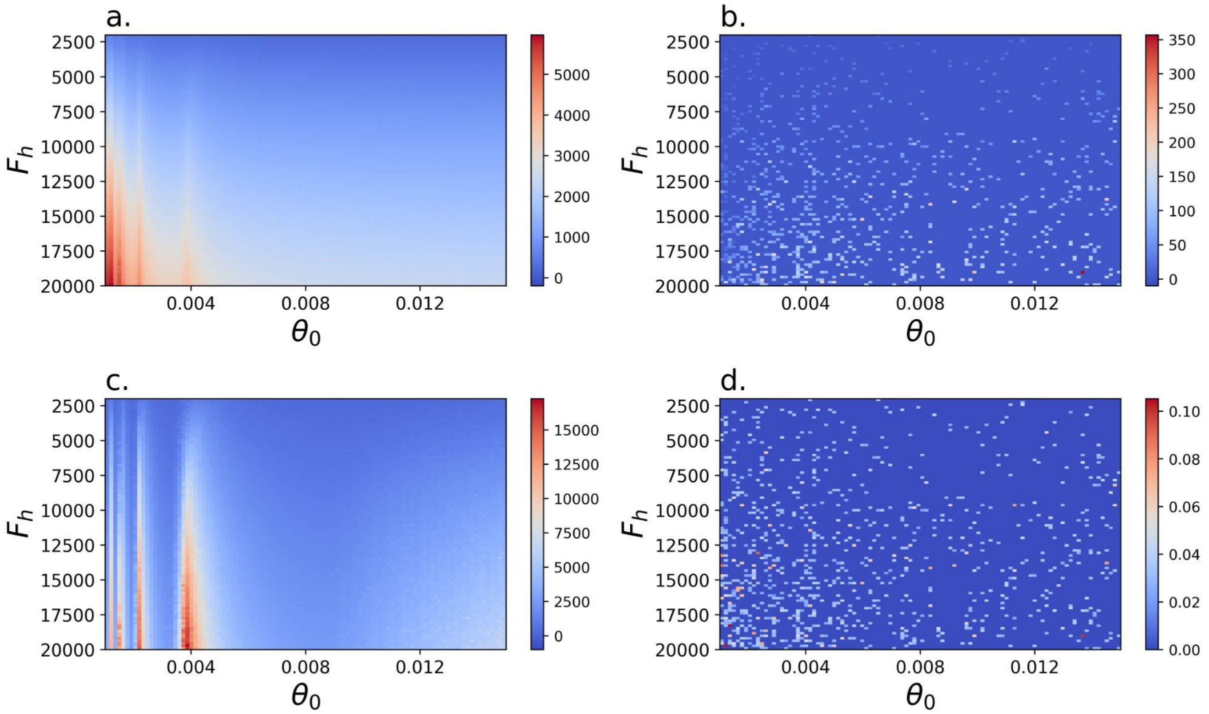

Figure S11 Impact of the rate of vaccination and initiation of low rate of transmission on model dynamics for p = 10^{-8} and exit from low transmission at F_l=F_h/8. The cumulative death rate from the **a**, wildtype and **b,** resistant strains, **c**, the number of wildtype-strain infected individuals at t_{v60}, the point in time when 60% of the population is vaccinated and **d**, the probability of resistant strain establishment.

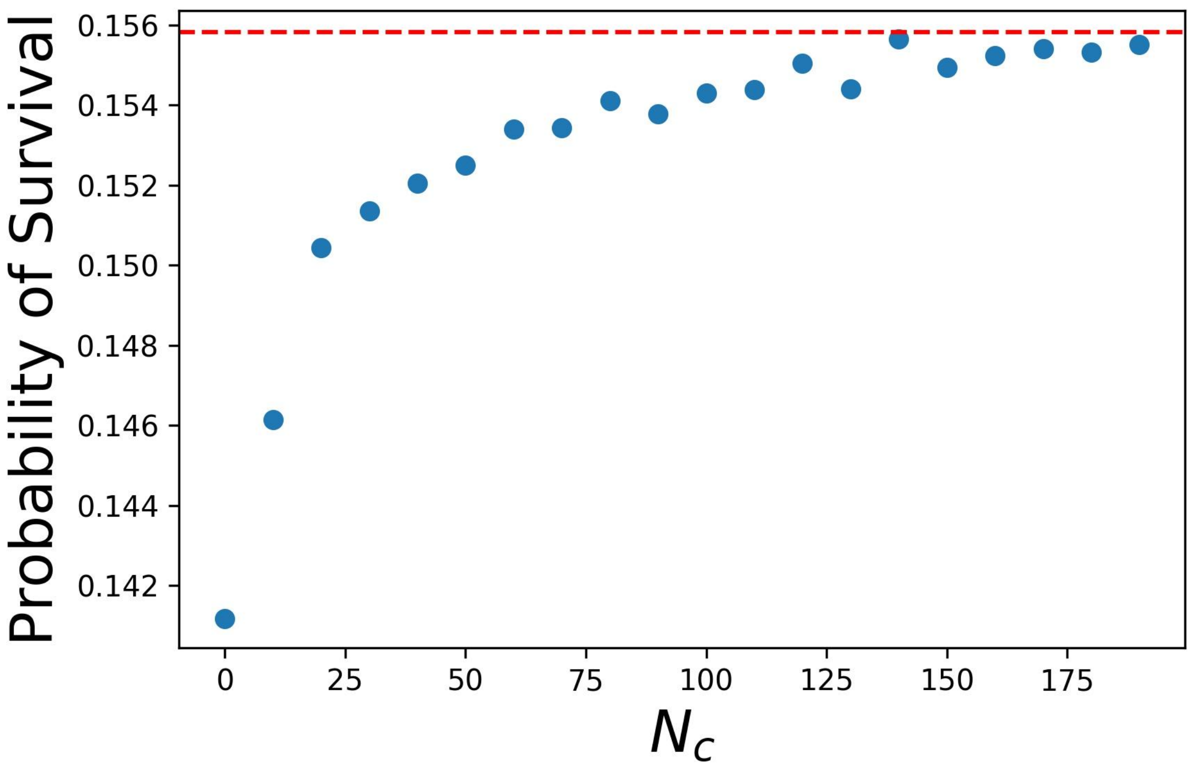

Figure S12. The fraction of surviving strains after $T=200$ days in 10^7 runs, first initialized with $I_{wt} = 200$ infected individuals. The red dashed line shows the expected fraction of surviving strains, as computed with **eq. 13**. The stochastic algorithm becomes exact, if no Tau Leaping is employed and instead the whole simulation is evaluated using the Gillespie SSA scheme.
